# Assessment of Hearing Impairment in Sick Newborns: A Prospective Observational Study

**DOI:** 10.7759/cureus.40457

**Published:** 2023-06-15

**Authors:** Jatin Nagal, Ramesh Choudhary, Mahendra Jain, Kailash Meena

**Affiliations:** 1 Department of Medical and Health, Government of Rajasthan, Jaipur, IND; 2 Department of Paediatrics, Juggilal-Kamlapat Lon (JK Lon) Hospital, Sawai Man Singh Medical College, Jaipur, IND; 3 Department of Neonatology, All India Institute of Medical Sciences, Bhopal, IND; 4 Department of Pediatrics, Sawai Man Singh Medical College, Jaipur, IND

**Keywords:** neonatal intensive care unit, automated auditory brainstem response, distortion-product otoacoustic emissions, otoacoustic emissions, hearing impairment

## Abstract

Background: Undiagnosed neonatal hearing loss causes severe language, cognitive, and behavioral problems in children. Sick newborns who spend 48 hours in the neonatal intensive care unit (NICU) have a 10- to 20-fold increased risk of permanent hearing loss. The aim of this study is to identify hearing impairment in high-risk sick newborns who spend at least 48 hours in the NICU.

Methods: The present prospective observational study was conducted at a single center. All sick neonates admitted to the NICU for a minimum of 48 hours at the JK Lon Hospital, Zanana Hospital, and Mahila Chikitsalaya, Sawai Man Singh (SMS) Medical College, Jaipur, Rajasthan, India, from June 2017 to May 2018 were considered eligible for the study. The primary outcome was the assessment of hearing loss of high-risk newborns using otoacoustic emissions at the time of discharge, six weeks of age, and automated auditory brainstem response (AABR) at three months of chronological age. The secondary outcome was to analyze the association of hearing loss with different risk factors in high-risk neonates.

Results: A total of 150 infants who had one or more risk factors were studied, 60 were female and 90 were male. No statistically significant difference in hearing loss was observed based on birth weight, sex, and gestational age. The first distortion-product otoacoustic emission (DPOAE) screening was done on infants at the time of discharge from the NICU. Eighty-three infants (55.33%) had “refer” on the first DPOAE and the remaining 67 (44.67%) were passed results at the time of discharge. At six weeks of life, on repeat screening with a second DPOAE test, 36% "refer" on the first screen had a "refer" result on the second DPOAE. However, 4.4% "pass" on the first screen turned out to be "refer" on the second screen. These 33 infants who had "refer" results on the second screen were subjected to testing. At 10 weeks of life, AABR was performed on 33 infants. Eleven infants out of 33 had sensorineural hearing loss (SNHL) on AABR. Hearing impairment with the DPOAE test was observed with risk factors neonatal hyperbilirubinemia (NHH), hypoxic ischemic encephalopathy (HIE), and very low birth weight (VLBW) and was statistically significant among all risk factors. But, no such association (between hearing impairment and risk factors) was observed with the AABR test. In our study, we found that the duration of mechanical ventilation in mean days 7.67±6.24 had statistically significant SNHL compared to the lesser duration of mechanical ventilation (p<0.001).

Conclusion: Two-stage DPOAE done prior to AABR is helpful in the early detection of hearing loss.

## Introduction

Early recognition of hearing loss (HL) and timely intervention is essential to prevent adverse speech and language outcomes due to permanent congenital or acquired HL in the neonatal period [1,2]. The first half of infancy is a critical period for early detection of HL and optimal intervention that would achieve normal speech and language development [3,4]. Newborn hearing screening is an important step through which this task can be achieved along with parenteral education and early intervention [5]. Universal hearing screening is needed to cover 7.5 million children worldwide, of whom 80% live in middle- to low-income countries [6,7]. About one-third of the worldwide population, mainly living in the developed world, received a complete newborn hearing screening that includes both healthy and sick newborns [8]. However, in resource-constrained countries with a high annual birth rate or a high number of home births, newborn hearing screening may be initiated with the at-risk newborn or the sick newborn (newborn with any medical or surgical condition) in the special newborn care unit (SNCU) or neonatal intensive care unit (NICU) [9]. In 2007, the United States Joint Committee on Infant Hearing (JCIH) Position Statement 2007 established risk factors for congenital permanent HL [10].

In 2006, the National Program for Prevention and Control of Hearing Loss was launched in India [6]. The program aims to detect severe-profound bilateral HL by the age of six months and rehabilitate it by the age of nine months. Currently, this program is implemented in many districts of India, human resources are trained in many districts, and awareness materials have been prepared and compiled for all centers [11].

Automated auditory brainstem response (AABR) and otoacoustic emission (OAE) tests are the best methods of hearing assessment among diagnostic methods because of their specificity and sensitivity. OAE assesses the function of the outer hair cells of the cochlea (pre-neural pathway) and is used in assessing cochlear diseases, such as exposure to viruses, noise, and ototoxic substances [12]. OAEs are broadly classified into two categories based on the use of external stimuli: spontaneous otoacoustic emission (SOAE) where no external stimuli are used, and evoked otoacoustic emission (EOAE) where external stimuli are used. EOAEs are further classified into three types: (1) a pure tone stimulus frequency OAE (SFOAE), (2) a click of tone burst stimuli generated using a transient-evoked OAE (TEOAE), and (3) two simultaneous pure tone stimuli (f1 and f2) produce distortion-product OAE (DPOAE) [13]. Compared with TEOAE, DPOAE is better because it is able to measure a greater frequency of the auditory sensitivity in terms of specific measurement as well as provide trustworthy information about frequency at 2 kHz or above. DPOAEs could also reduce the false positive rate caused by the vernix in the ear canal, transient middle ear pathology, or fetal fluid in the ear canal, especially at the first 24-48 hours of life by passing neonates with genuine cases of permanent HL below 50 dB. DPOAE was also shown to have a good correlation with both audiometric thresholds and AABR findings, and DPOAE is now widely applied in newborn hearing screening programs [14]. The present observational study was conducted to identify hearing impairment in high-risk sick newborns who spend at least 48 hours in the NICU. 

## Materials and methods

This prospective observational, single-center study was conducted from June 2017 to May 2018 in the NICU of J.K. Lon Hospital, Zanana Hospital, and Mahila Chikitsalaya, Sawai Man Singh (SMS) Medical College, Jaipur, Rajasthan, India. All sick newborns ≥28 weeks of gestational age who remained in the NICU for at least 48 hours were considered eligible for the study. The following neonates were excluded from the study: newborns who stayed in the NICU for <48 hours; newborns <28 weeks of gestational age; newborns with defects of auricles, auditory canal, or other life-threatening anomalies; neonates who died or did not complete all phases of the study; or parents who refused consent. Informed written consent for the study was obtained from the parent for enrollment in the study after explaining the purpose of the study. They were also provided with a participant information sheet. The Institute Research Review Board of SMS Medical College and the attached hospital (letter number 17502/2017, thesis no. 98912) approved the study. A routine otorhinolaryngologic examination of all participants was carried out, which consisted of an inspection of the pre-aural, pinna, and post-aural regions in the ear, nose, and throat department. Occluding wax or debris was gently removed using a cotton-tipped swab. A detailed history, complete clinical examination, and general examination were carried out, and potential risk factors were identified. Neonates underwent hearing assessment at the time of discharge using the DPOAE test. The machine used for this test was the “Echo Screen UC-232 S/N: 251430” by Natus Europe GmbH (Planegg, Germany). Otoacoustic emission probe calibration was carried out daily to ensure the infants were screened with a functioning probe. During the measurement, two pure tone stimuli (f1 and f2), where f2 was higher than f1, were presented with an f1/f2 ratio of approximately 1.22 (range 1.21-1.23) to obtain a robust DPOAE response in human ears [7]. With the probe tip in place and the check fit procedure passed, DPOAEs were initiated. Testing was performed in an otorhinolaryngologic audiometry noise-isolation room near the NICU at the time of discharge from the hospital.

Testing was done while the infant was naturally sleeping or alert and calm or under sedation (20 mg/kg of triclofos given), as the results of OAE are not affected by sedation. The test results were displayed on the screening unit as a "pass" or a "refer.” This was recorded in the book of vaccination and the newborn medical record. The results of OAE were interpreted according to the Rhode Island criteria: (1) pass: response is 3 dB or more in at least four frequency bands (2000, 2500, 3200, and 4000 Hz) and (2) “refer”: no response is present in any frequency band [12]. An infant with a "refer” or "pass" result at six weeks of age at the time of vaccination was followed up and a second DPOAE was performed at that time. Participants had a second DPOAE to check for acquired HL due to drugs, mechanical ventilation (MV), hypoxic ischemic encephalopathy (HIE), or sepsis [15]. Auditory brainstem response audiometry (AABR) was performed at 14 weeks of age at the time of vaccination in infants who were "referred" during a second OAE screening. Sensorineural HL (SNHL) in children (0-14 years) is defined as HL >30 dB in the better-hearing ears. The severity of HL was classified into six categories: slight HL, mild HL, moderate HL, moderate-to-severe (or moderately severe) HL, severe HL, and profound HL where HL in the better ear is more than 16-25 dB, 26-40 dB, 41-55 dB, 56-70 dB, 71-90 dB, and >90 dB, respectively [16]. The data collected were subjected to analysis and their correlation with the risk factors. Infants with abnormal AABR were referred for follow-up for auditory and language development. We analyzed quantitative data using the "z"-test and qualitative data using the chi-square test and chi-square test with Yates's correction.

The sample size was calculated at a 95% confidence level, assuming first-stage failure by OAE in 6.1% of high-risk neonates as per the results of the reference article. At the absolute allowable error of 4% (precision), a minimum of 138 high-risk neonates are required as a sample size, which is further enhanced and rounded off to 150 patients expecting 10% dropout/loss to follow-up/attrition. 

## Results

**Figure 1:**
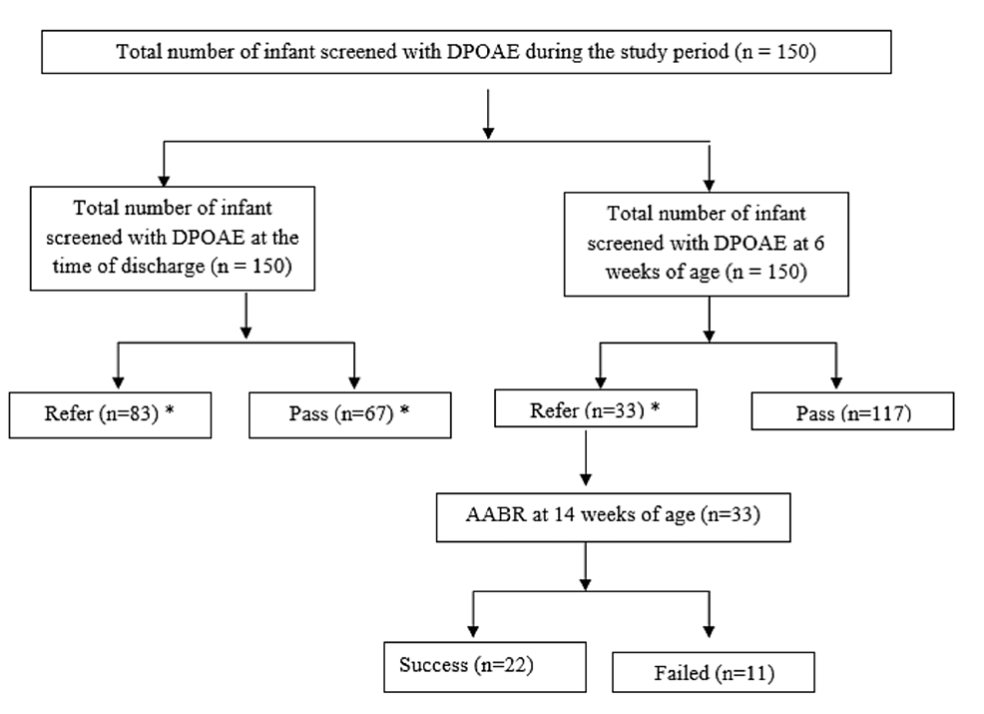
Participant flow diagram. *30 infants who were "refer" at the time of discharge remained "refer" at six weeks of age and three out of 67 infants who were "pass" at the time of discharge came out "refer" at six weeks of age during DPOAE hearing screening. DPOAE: distortion-product otoacoustic emission; AABR: automated auditory brainstem response.

Figure 1 depicts the flow diagram of participant recruitment in this study. A total of 150 newborns were screened during the study period by DPOAE at the time of discharge and at six weeks of age. In DPOAE at the time of discharge, 83 newborns (55.33%) had “refer” in either or both ears and 67 (44.65%) newborns were passed in both ears. Among 83 (55.33%), 55 were preterm and 28 were term. Similarly, among 67 (44.67%) “pass” infants, 39 (26.00%) were preterm and 28 were term. At six weeks of age, among 33 (22.00%) who were “refer” on OAE, 21 (14.00%) were preterm and 12 (8.00%) were term infants. Among 117 (78.00%) “pass” infants on OAE at six weeks, 73 (48.67%) infants were preterm and 44 (29.33%) were term. Thirty (20.00%) infants who were "refer" at the time of discharge remained "refer" at six weeks on OAE. Three (2.00%) out of 67 (44.67%) who were "pass" at the time of discharge came out to be "refer" at six weeks. AABR was conducted on 33 infants who failed screening at six weeks of age, 11 infants out of 33 were having SNHL on AABR. In our study, of a total of 150 infants, 90 (60%) were males and 60 (40%) were females. The baseline characteristics of enrolled newborns are summarized in Table 1.

**Table 1:** Baseline characteristics of study participants. #Data presented as number (percentage). NICU: neonatal intensive care unit.

Baseline characteristics		No. of sick infants who spends minimum 48 hours in NICU^#^
Gender	Male	90 (60)
	Female	60 (40)
Gestational age	28-33 weeks	48 (32)
	33-37 weeks	46 (30.67)
	≥37 weeks	56 (37.33)
Birth weight	<1000 g	1 (0.67)
	1000-1499 g	33 (22.00)
	1500-2499 g	65 (43.33)
	≥2500 g	51 (34.00)

Study outcomes across major risk factors for HL are summarized in Tables 2, 3. No significant differences were observed in our study during screening at discharge based on gestational age, birth weight, sex, MV duration, HIE, and "hyperbilirubinemia not requiring exchange transfusion." Hearing impairment with the DPOAE test was observed to have a statistically significant association with risk factors such as the use of amikacin, preterm, very low birth weight (VLBW) infants, and "neonatal hyperbilirubinemia requiring exchange transfusion." At six weeks of age, "hyperbilirubinemia requiring exchange transfusion" and HIE were significantly associated with HL compared to other sick newborns. However, on testing with AABR, no such association was seen. In our study, we found that those infants who had MV for mean of 7.67±6.24 days had SNHL compared to those who had MV for a lesser duration and had no HL.

**Table 2:** Causes of NICU admission associated with hearing impairment by two-stage DPOAE. NICU: neonatal intensive care unit; DPOAE: distortion-product otoacoustic emission; HIE: hypoxic ischemic encephalopathy; DVET: double volume exchange transfusion; NNH: neonatal hyperbilirubinemia; MV: mechanical ventilation.

Variables		DPOAE at the time of discharge			DPOAE at six weeks of age		
		Pass	Refer	p-value	Pass	Refer	p-value
Gestational age	Gestational age <37 weeks	38 (40.42)	56 (59.57)	0.08	73 (77.66)	21 (22.34)	0.23
	Gestational age ≥37 weeks	27 (48.21%)	29 (51.79%)		43 (76.79)	13 (23.21)	
	Mean gestational age	34.63±2.94	34.00±3.00	0.91	34.21±2.98	34.47±3.02	0.86
Gender	Male	41 (45.56)	49 (54.44)	0.37	68 (75.56)	22 (24.44)	0.48
	Female	24 (40.00)	36 (60.00)		48 (80.00)	12 (20.00)	
Birth weight	<1000 g	0 (0.00)	1 (100.00)	0.001	1 (100.00)	0 (0.00)	0.09
	1000-1499 g	11 (33.33)	22 (66.67)		24 (72.73)	9 (27.27)	
	1500-2499 g	29 (44.61)	36 (55.38)		54 (83.08)	11 (16.92)	
	≥2500 g	25 (49.02)	26 (50.98)		37 (72.55)	14 (27.45)	
	Mean birth weight	2.12±0.70	2.02±0.68	0.31	2.05±0.68	2.11±0.73	0.29
Ototoxic drug amikacin	Given	41 (64.05%)	49 (57.65%)	0.18	73 (78.89)	17 (21.11)	0.07
	Not given	24 (37.50%)	36 (42.35%)		43 (70.00)	17 (30.00)	
	Mean drug duration	8.07±3.47	9.83±4.51	0.0069	8.78±3.90	9.92±4.90	0.39
MV	≤7 days	6 (28.57)	15 (71.43)	0.45	11 (52.38)	10 (47.62)	0.92
	>7 days	0 (0.00)	2 (100.00)		1 (50.00)	1 (50.00)	
	Mean MV duration	1.83±0.69	4.47±3.44	0.11	3.67±2.09	3.91±4.08	0.84
Perinatal asphyxia	HIE	12 (38.71)	19 (61.29)	0.73	21 (67.74)	10 (32.25)	0.001
	No HIE	53 (44.54)	66 (55.46)		95 (79.83)	24 (20.17)	
NNH	DVET	0 (0.00)	5 (100.00)	0.001	1 (20.00)	4 (80.00)	0.001
	Phototherapy	9 (34.61)	17 (65.38)	0.62	22 (84.62)	4 (15.38)	0.71
	No NNH	56 (47.06)	63 (52.94)		93 (78.15)	26 (21.85)	

**Table 3:** Causes of NICU admission associated with SNHL by ABER. NICU: neonatal intensive care unit; ABER: auditory brain stem-evoked response; HIE: hypoxic ischemic encephalopathy; DVET: double volume exchange transfusion; SNHL: sensorineural hearing loss; NNH: neonatal Hyperbilirubinemia; MV: mechanical ventilation.

Variables		ABER at 14 weeks of age (n=33)		p-value
		Success (n=22)	Fail (n=11)	
Gender	Male	13 (61.90)	8 (38.10)	0.12
	Female	9 (75.00)	3 (25.00)	
Birth weight	<1000 g	0 (0.00)	0 (0.00)	0.46
	1000-1499 g	8 (88.89)	1 (11.11)	
	1500-2499 g	5 (50.00)	5 (50.00)	
	≥2500 g	9 (64.29)	5 (35.71)	
Gestational age	<37 weeks	13 (65.00)	7 (35.00)	0.71
	≥37 weeks	9 (69.23)	4 (30.77)	
Ototoxic drug amikacin	Given	15 (65.22)	8 (34.78)	0.34
	Not given	7 (70.00)	3 (30.00)	
	Drug duration	11.00±5.33	7.75±3.19	0.69
MV	Duration	2.00±0.00	7.67±6.24	<0.01
	≤7days	8 (80.00)	2 (20.00)	<0.01
	>7 days	0 (0.00)	1 (100.00)	
Perinatal asphyxia	HIE	8 (80.00)	2 (20.00)	0.26
	No HIE	14 (60.87)	9 (39.13)	
NNH	DVET	1 (25.00)	3 (75.00)	0.07
	Phototherapy	1 (33.33)	2 (66.67)	
	No NNH	20 (76.92)	6 (23.08)	

## Discussion

This prospective observational cohort study was performed to assess HL in sick infants by using DPOAE at discharge and at six weeks of age, followed by AABR performed at 14 weeks of age in those DPOAE failing (refer) at six weeks of age. In our study, 7.33% of infants had SNHL. In contrast to our study, other previously published studies had 2%-6.5% SNHL, but these studies were performed either in high-risk neonates or healthy and high-risk neonates [15-18].

Gouri et al. studied hearing assessment on healthy and sick newborns with "AABR after transient-evoked otoacoustic emissions (TEOAE)," where 137 out of 415 newborns required NICU admission [15]. In their study, 8.03% of total NICU admissions had SNHL, which is almost comparable to our study; otherwise, the overall prevalence of SNHL was 4.3% [15]. Pourarian et al. did a similar study in NICU-admitted sick neonate and their study prevalence was found to be 13.7% by applying the two-stage method AABR after TEOAE [16]. Stadio et al. found a prevalence of SNHL to be 7.8% using two-stage hearing screening, i.e., "AABR after TEOAE," which was almost similar to our study [17]. Hardani et al. conducted a study with a screening protocol of the first TEOAE in all sick neonates, then a second TEOAE in those sick neonates who failed the first TEOAE, not all sick neonates, followed by AABR and hearing auditory stable-state response (ASSR) tests [18]. In their study, the prevalence of total HL was 5.09% and the SNHL prevalence was only 2.64%; in contrast, we performed DPOAE two times in all sick neonates.

In contrast, in our study, we found 1%-1.5% more SNHL that could have been missed by two-step hearing screening. In our study, we found VLBW infants, amikacin, HIE, and neonatal hyperbilirubinemia (NNH) required for exchange transfusion caused more hearing impairment (p-value <0.5) than other diseases during DPOAE screening. However, on testing with AABR, no such association was seen. In our study, we found that those infants who had MV for a mean of 7.67±6.24 days had SNHL compared to those who had MV for a lesser duration and had no HL. Pourarian et al. found that oxygen therapy and the use of antibiotics were statistically significant compared to other risk factors including the risk factor "duration of MV of more than five days" [16]. On the contrary, in our study, the mean duration of MV (7.67±6.24 days) was associated with statistically significant SNHL compared to a shorter duration. Khairy et al. studied HL among neonates admitted to the NICU, and their findings were that MV for more than five days and the use of ototoxic drugs were statistically significant risk factors for HL compared to other risk factors [19]. 

Limitation of study

The study incorporated fewer risk factors as compared to the factors suggested by the Joint Commission on Infant Hearing (JCIH). Moreover, this is a single-center study, and it is advocated that such type of trials should be conducted in other centers for better availability of results. Additionally, we have a smaller sample size, and a much larger sample size may reveal better results. Furthermore, a significant analysis of hearing screening would require studies with larger sample sizes. Our 100% follow-up is the strength of our study. 

## Conclusions

For early detection of HL, "two-stage DPOAE preceded by AABR" can be successfully implemented as a newborn hearing screening program in a hospital setting. Minimizing mechanical ventilation may improve HL in sick newborns.
